# In Vitro Identification of New Transcriptomic and miRNomic Profiles Associated with Pulmonary Fibrosis Induced by High Doses Everolimus: Looking for New Pathogenetic Markers and Therapeutic Targets

**DOI:** 10.3390/ijms19041250

**Published:** 2018-04-20

**Authors:** Simona Granata, Gloria Santoro, Valentina Masola, Paola Tomei, Fabio Sallustio, Paola Pontrelli, Matteo Accetturo, Nadia Antonucci, Pierluigi Carratù, Antonio Lupo, Gianluigi Zaza

**Affiliations:** 1 Renal Unit, Department of Medicine, University of Verona, Piazzale Stefani 1, 37126 Verona, Italy; simona.granata@univr.it (S.G.); gloria.santoro@univr.it (G.S.); valentina.masola@unipd.it (V.M.); paola.tomei@univr.it (P.T.); nadia.antonucci@univr.it (N.A.); antonio.lupo@univr.it (A.L.); 2 Department of Emergency and Organ Transplantation, University of Bari “Aldo Moro”, Piazza Giulio Cesare 11, 70124 Bari, Italy; fabio.sallustio@uniba.it (F.S.); paola.pontrelli@uniba.it (P.P.); accetturo.m@gmail.com (M.A.); 3 Department of Basic Medical Sciences, Neuroscience and Sense Organs, University of Bari “Aldo Moro”, Piazza Giulio Cesare 11, 70124 Bari, Italy; 4 Department of Respiratory Diseases, University of Bari “Aldo Moro”, Piazza Giulio Cesare 11, 70124 Bari, Italy; pierluigi.carratu@uniba.it

**Keywords:** epithelial to mesenchymal transition, mTOR inhibitor, pulmonary fibrosis, transcriptomics, miRNome, everolimus

## Abstract

The administration of Everolimus (EVE), a mTOR inhibitor used in transplantation and cancer, is often associated with adverse effects including pulmonary fibrosis. Although the underlying mechanism is not fully clarified, this condition could be in part caused by epithelial to mesenchymal transition (EMT) of airway cells. To improve our knowledge, primary bronchial epithelial cells (BE63/3) were treated with EVE (5 and 100 nM) for 24 h. EMT markers (α-SMA, vimentin, fibronectin) were measured by RT-PCR. Transepithelial resistance was measured by Millicell-ERS ohmmeter. mRNA and microRNA profiling were performed by Illumina and Agilent kit, respectively. Only high dose EVE increased EMT markers and reduced the transepithelial resistance of BE63/3. Bioinformatics showed 125 de-regulated genes that, according to enrichment analysis, were implicated in collagen synthesis/metabolism. Connective tissue growth factor (*CTGF*) was one of the higher up-regulated mRNA. Five nM EVE was ineffective on the pro-fibrotic machinery. Additionally, 3 miRNAs resulted hyper-expressed after 100 nM EVE and able to regulate 31 of the genes selected by the transcriptomic analysis (including *CTGF*). RT-PCR and western blot for *MMP12* and *CTGF* validated high-throughput results. Our results revealed a complex biological network implicated in EVE-related pulmonary fibrosis and underlined new potential disease biomarkers and therapeutic targets.

## 1. Introduction

Everolimus (EVE), marketed as Certican, is a pharmacological agent widely used in the anti-rejection therapy of solid organ transplantation and in the treatment of certain tumors (e.g., in advanced renal cell carcinoma, subependymal giant cell astrocytoma associated with tuberous sclerosis, pancreatic neuroendocrine tumors, breast cancer) [1]. Similar to Sirolimus and Tamsilorimus, it exerts its immunosuppressive activity by inhibiting mammalian target of rapamycin (mTOR), a phosphoinositide 3-kinase-related protein that controls cell cycle, protein synthesis, angiogenesis and autophagy [2]. These important multi-factorial biological/cellular effects allow this drug to avoid/minimize the onset of acute rejection episodes and to slow down the progression of chronic allograft lesions [3,4].

However, some authors have reported a high rate of discontinuation secondary to side effects after the introduction of this drug [5,6,7]. Among them, pneumonitis or interstitial lung disease with a range of pulmonary histopathologic changes (including alveolar hemorrhage, pulmonary alveolar proteinosis, focal fibrosis, bronchiolitis obliterans organizing pneumonia) have been largely reported in clinical records and they have been associated with worsened patients’ clinical outcomes and drug discontinuation [8,9,10,11,12,13,14,15,16]. The incidence of this complications is 2–11%, frequently reported between 1 and 51 months after the beginning of mTOR inhibitor therapy [17,18,19].

The pathogenic mechanism underlying lung toxicity is multi-factorial and epithelial to mesenchymal transition (EMT) of airway cells seems to have a pivotal role [20,21,22,23]. Our group has recently demonstrated that high doses of EVE are associated with a reprogramming of gene expression in several epithelial cell lines (airway, renal epithelial proximal tubular and hepatic cells) with a consequent loss of their phenotype (junctions and apical-basal polarity) and the acquisition of mesenchymal traits increasing the motility and enabling the development of an invasive and pro-fibrotic phenotype [24,25,26].

High dosage of EVE eliminating negative crosstalk from mTORC1/S6K, leads to activation of mTORC2 that enhances AKT phosphorylation at Ser473 and stimulates PI3K-AKT signaling that induces renal fibrosis [26,27,28,29,30].

The pro-fibrotic attitude of EVE has also been confirmed in vivo in renal transplant patients through the estimation of an arbitrary pulmonary fibrosis index score in renal transplant patients chronically treated with this drug. In this patients’ subset, high blood trough level of EVE was associated with a high rate of pulmonary signs of fibrosis [24].

However, although the aforementioned studies and the large clinical evidences, the complete biological machinery involved in this condition has not been completely clarified.

Therefore, we employed, for the first time, a highthroughput approach combining a transcriptomic with a miRNome analysis to study the capability of EVE to induce pro-fibrotic changes in primary bronchial epithelial cells.

All together our results could represent a step forward in the comprehension of the mTOR-I associated biological machinery and in the identification of new targets for therapeutic interventions.

## 2. Results

### 2.1. High Dosage Everolimus (EVE) Induced Epithelial to Mesenchymal Transition (EMT) of BE63/3 (Primary Bronchial Epithelial Cells)

To confirm our previous results obtained in immortalized bronchial and pulmonary cell lines [24], we decided to measure by Real Time-PCR the expression level of alpha smooth muscle actin (*α-SMA)*, vimentin (*VIM*), and fibronectin (*FN*) in BE63/3 treated for 24 h with 2 different dosages of EVE (5 and 100 nM) chosen according to literature evidences [31,32,33,34] and previous experiments performed by our research group in different cell lines [24,25,26].

**Figure 1:**
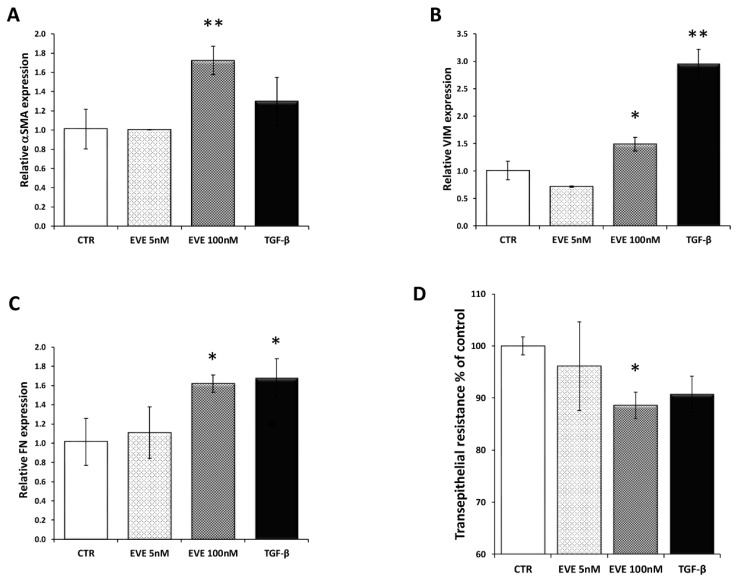
Gene expression of epithelial to mesenchymal transition (EMT) related markers. Relative (**A**) alpha smooth muscle actin (*α-SMA*), (**B**) fibronectin (*FN*) and (**C**) vimentin (*VIM*) expression evaluated by Real-time PCR in BE 63/3 cells treated or untreated with Everolimus (EVE) (5 and 100 nM) or TGF-β (20 ng/mL); expression values were normalized to glyceraldehyde-3-phosphate dehydrogenase (GAPDH). Mean ± S.D. (error bars) of three separate experiments performed in triplicate. * *p* < 0.05, ** *p* < 0.01 vs. control (CTR). (**D**) Histogram represents transepithelial resistance as percentage change with respect to control cells. * *p* < 0.05 vs. CTR.

Only high dose of EVE (100 nM), similarly to TGF-β (20 ng/mL), increased the mRNA level of the EMT-related markers (Figure 1A–C). Moreover E-cadherin resulted downregulated although it did not reach a statistically significant level (Figure S1). Contrarily, 5 nM EVE was ineffective (Figure 1A–C).

Additionally, high dosage of EVE was also able to reduce the transepithelial resistance (TER) evaluated by a Millicell-ERS ohmmeter indicating dysfunctional tight junctions (Figure 1D).

### 2.2. Transcriptomic Analysis Revealed That High Dosage of EVE Up-Regulated Genes Involved in Collagen Synthesis and Metabolism

Gene expression profiling evaluated by transcriptomic analysis revealed that in vitro treatment of BE63/3 cells with 100 nM EVE for 24 h deregulated 147 probe sets (corresponding to 125 genes): 60/147 probe sets (47 genes) resulted up-regulated while 87/147 probe sets (corresponding to 78 genes) were down-regulated (≥1.5-fold change) in EVE-treated cells compared with control (CTR) (Table 1). According to enrichment analysis, selected genes belonged to 44 pathways (Table 2) and 5 of them were involved in collagen synthesis/metabolism and regulation of stress fiber assembly. Interestingly, connective tissue growth factor (*CTGF*) was a representative gene in all these pro-fibrotic pathways.

Instead, low dosage EVE (5 nM) was able to change the expression level of only 33 probe sets (24 genes): 25/33 probe sets (20 genes) were hyper-expressed and 4 probe sets (4 genes) down-regulated after treatment (Table 3). None of the selected pathways was associated with the pro-fibrotic cellular machinery (Table 4).

**Figure 2:**
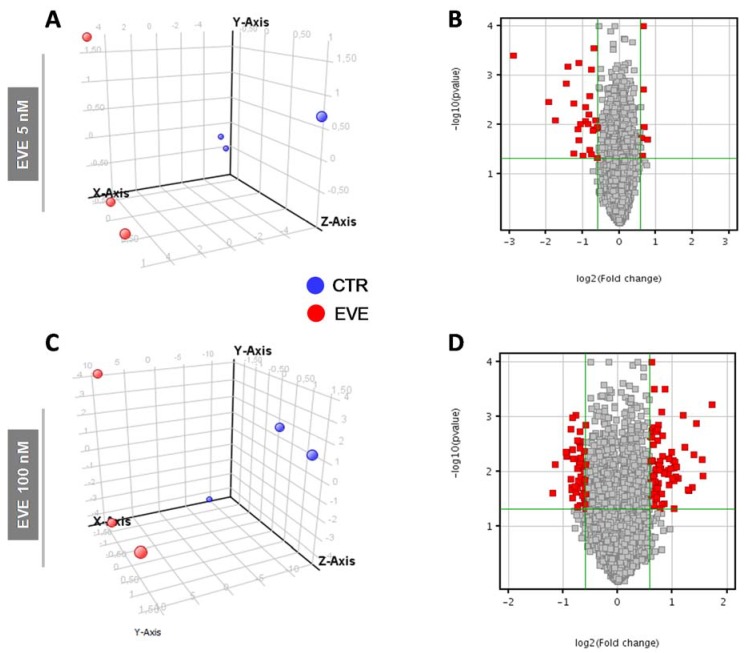
Principal Component Analysis (PCA) and Volcano Plot discriminating BE63/3 CTR from EVE treated cells. PCA plots were built using the expression level of all differentially expressed genes obtained from mRNA expression profiling after treatment with (**A**) 5 nM and (**C**) 100 nM EVE. Volcano Plot based on fold change (Log2) and p value (−Log10) of all genes identified in BE63/3 after treatment with (**B**) 5 nM and (**D**) 100 nM EVE. In both graphs red circles indicate the genes that showed statistically significant change.

Principal component analysis (PCA) and volcano plot showed the degree of separation of untreated versus treated cells at both EVE dosages (Figure 2).

### 2.3. MiRNome Analysis Identified Specific MicroRNAs Deregulated by EVE

To gain insights into the mechanism leading to EMT induced by EVE and to discover possible regulatory miRNAs of this effect, we performed a miRNome analysis by miRNA Complete Labeling and Hybridization kit. Statistical analysis identified three miRNAs up-regulated after high dosage (100 nM) (Table 5) and four after treatment with EVE at low dosage (5 nM) (Table 6). Among these, miR-8485 was the most up-regulated miRNA (more than 4-fold changes in both treatments).

By matching mRNA and miRNA expression data, we found that 31 genes were specific target of the three identified miRNAs (Table 7).

### 2.4. Gene Expression and Protein Analysis for Matrix Metalloproteinase 12 (MMP12) and Connective Tissue Growth Factor (CTGF) Validated High-Throughput Results

**Figure 3:**
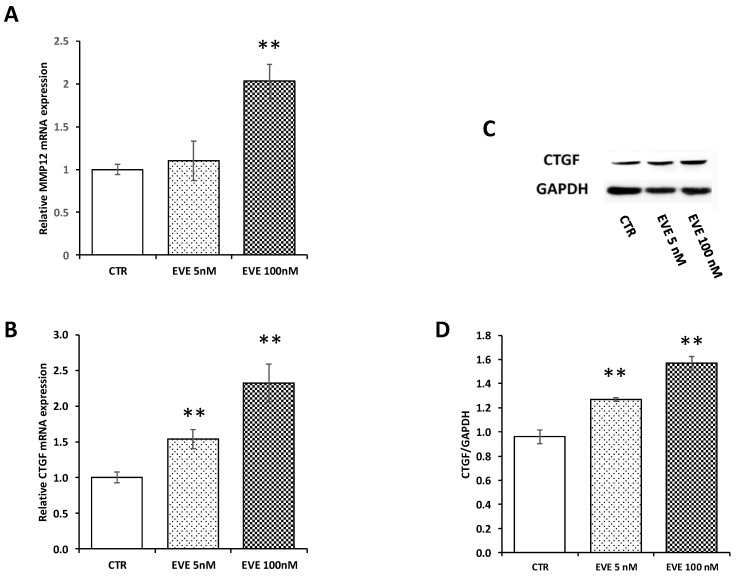
Gene expression of *MMP12* and connective tissue growth factor (*CTGF*). mRNA level of (**A**) *MMP12* and (**B**) *CTGF* evaluated by real-time PCR in BE63/3 cells treated or not with EVE (5 and 100 nM). Data were normalized to GAPDH expression. Mean ± SD (error bars) of two separate experiments performed in triplicate. ** *p* < 0.001, * *p* < 0.05 vs. CTR. (**C**) Representative western blotting experiments for *CTGF*. (**D**) Histogram represents the mean ± SD of *CTGF* protein level. *GAPDH* was included as loading control. ** *p* < 0.001 vs. CTR.

In order to validate microarray results, we measured by Real-Time PCR the level of mRNA expression of *MMP12* and *CTGF*. Both transcripts were up-regulated after treatment with 100 nM EVE. Contrarily 5 nM EVE had no effect (Figure 3A,B). In addition, western blot analysis of *CTGF* confirmed gene expression results at protein level (Figure 3C,D).

### 2.5. Validation of Transcriptomic Results in an Additional Primary Cell Line (BE121/3)

**Figure 4:**
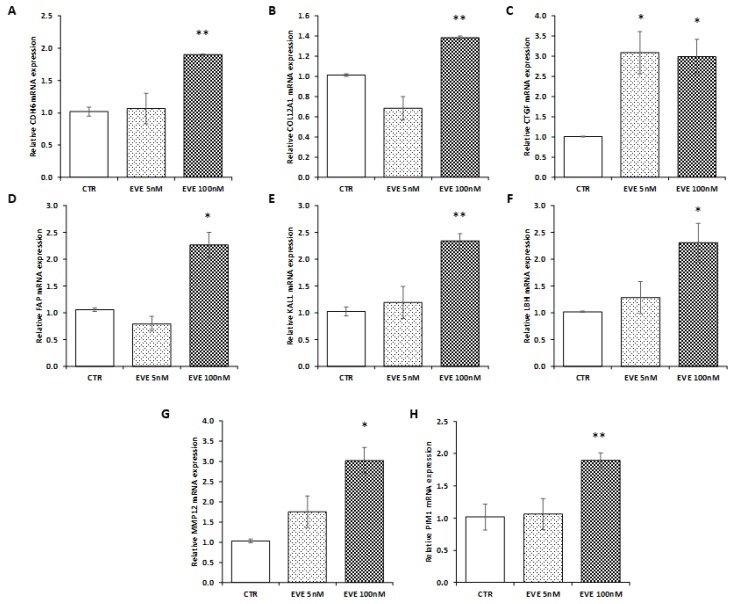
Gene expression in BE121/3. mRNA level of (**A**) *CDH6*, (**B**) *COL12A1*, (**C**) *CTGF*, (**D**) *FAP*, (**E**) *KAL1*, (**F**) *LBH*, (**G**) *MMP12*, (**H**) *PIM1* evaluated by real-time PCR in BE121/3 cells treated or not with EVE (5 and 100 nM). Data were normalized to *GAPDH* expression. Mean ± SD (error bars) of two separate experiments performed in triplicate. ** *p* < 0.001, * *p* < 0.05 vs. CTR.

To confirm transcriptomic results, we decided to measure the expression level of 8 selected genes (involved in EMT) up-regulated after high dosage EVE in a new primary bronchial epithelial cell line. As showed in Figure 4, results were in line with those obtained in BE63/3 (Figure 4).

### 2.6. High Dosage EVE Up-Regulated CTGF and Collagen1 in Fibroblasts and Hepatic Stellate Cells

To validate the pro-fibrotic effect of high dosage EVE we measured the expression level of collagen1 and CTGF in NIH/3T3 (mouse embryo fibroblast cell line) treated with EVE.

**Figure 5:**
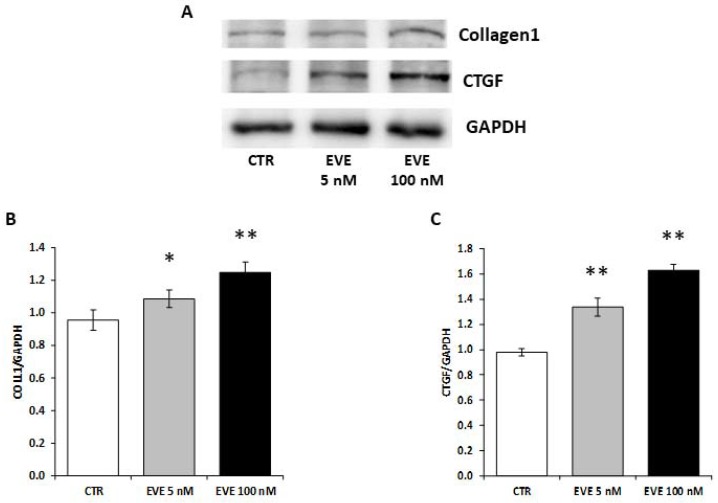
Protein levels of collagen1 and CTGF in NIH/3T3 cells. (**A**) Representative western blotting experiments for collagen1 and CTGF. Histograms represent the mean ± SD of (**B**) collagen1 and (**C**) CTGF protein levels. GAPDH was included as loading control. ** *p* < 0.001, * *p* < 0.05 vs. CTR.

Interestingly, also in fibroblasts high dosage EVE up-regulated the protein levels of collagen1 and CTGF (Figure 5).

Also, in hepatic stellate cells high dosage EVE induced the up-regulation of CTGF and collagen1 (Figure S2).

## 3. Discussion

Pulmonary fibrosis is a potential serious adverse effect following administration of mTOR-I in patients undergoing solid organ transplantation or receiving anti-cancer therapies. It is generally accepted that pulmonary disease is related to mTOR-I therapy, whether the following conditions are present: (1). The symptoms of pulmonary disease occur after initiation of mTOR-I therapy; (2). Infection, other pulmonary diseases or toxicity associated with other drugs are excluded; (3). mTOR-I minimization or discontinuation lead to resolution of the symptoms. In fact, the dose-dependent effect was proved by the observation of this disease particularly in patients receiving high doses of mTOR-I.

Pulmonary manifestations in these patients are numerous and include several clinical/histological phenotypes (e.g., focal pulmonary fibrosis, bronchiolitis obliterans with organizing pneumonia) [8,9,35,36].

This multi-factorial and heterogeneous clinical condition is often responsible for drug discontinuation and it requires long and expensive clinical evaluations and treatments (e.g., antibiotics, corticosteroids, immunosuppressive drugs) [14] with the involvement of a multidisciplinary team of experts (e.g., pulmonologists, infectivologists, nephrologists).

The etiopathogenic mechanism of pulmonary toxicity associated with mTOR-I therapy is not known and several in vivo and in vitro studies have tried to define the underlying mechanisms. It has been proposed a T cell-mediated autoimmune response induced when pulmonary cryptic antigens are exposed, leading to lymphocytic alveolitis and interstitial pneumonitis [15]. Other possible pathogenic mechanisms could be a delayed-type hypersensitivity reaction [9] or pulmonary inflammation as a direct effect of mTOR-I to stimulate cells of the innate immune system to produce proinflammatory cytokines [37,38].

Additionally, Ussavarungsi et al. have reported that sirolimus may induce granulomatous interstitial inflammation and proposed a mechanism of T-cell mediated hypersensitivity reaction triggered by circulating antigens or immune complexes in the lungs [39].

Moreover, several authors have emphasized the pathogenetic role of the EMT of bronchial epithelial cells in these important Everolimus (EVE)-related adverse events [20,21,22,23].

To obtain more insights, we decided to employ, for the first time, innovative high throughput technologies, to identify new elements involved in the biological/cellular reprogramming induced by high dose of mTOR-I and leading to fibrosis.

In vitro experiments using classical bio-molecular strategies, confirmed, in primary bronchial epithelial cell lines, our previous results demonstrating the ability of high dosages EVE to induce EMT. In particular, 100 nM EVE caused the up-regulation of EMT-related genes (*α-SMA, VIM, FN*) and reduced the trans-epithelial resistance to the same levels induced by TGF-β. Then, high doses of this drug significantly changed the expression level of 125 genes (47 up- and 78 down-regulated).

Several of the selected genes were target of miR-8485, the top significant and up-regulated microRNA (miRNA) by EVE 100 nM. Other 2 miRNAs were identified after the same treatment: miR-937-5p and miR-5194. Except for miR-8485, at our knowledge, none of them has been previously associated with fibrosis or supposed to be regulatory of genes implicated in this process. It’s unquestionable that further studies are warranted to confirm the involvement of these miRNAs in EVE induced EMT since all identified miRNAs were up-regulated demonstrating their possible role as enhancer of fibrotic machinery. This could be in line with recent findings suggesting that miRNA-mediated down-regulation is not a one-way process and some miRNAs could up-regulate gene expression in specific cell types and conditions with distinct transcripts and proteins [40,41]. It is noteworthy that these miRNAs are up-regulated also after treatment with 5 nM EVE. Many reasons could be responsible of this effect. In particular, the expression of these miRNAs could be regulated by several factors and networks (some of them also unrelated to mTOR-I treatment). Additional studies are needed to clarify the role of miRNA in EVE-mediated pro-fibrotic effect.

Moreover, analyzing the results of the transcriptomic analysis and the hypothetic targets of miR-8485, we found that connective tissue growth factor (*CTGF*), a protein secreted into the extracellular environment where it interacts with distinct cell surface receptors, growth factors and extra-cellular matrix [42,43] was one of the top scored genes. Gene expression by RT-PCR and protein analysis by western blotting confirmed the result obtained by microarray.

It is well known that CTGF modulates the activities of TGF-β or vascular endothelial growth factor (*VEGF*), with consequent pro-fibrotic and angiogenetic effects [44,45,46,47]. However, the overexpression of CTGF in fibroblast of mice caused tissue fibrosis in vivo [48] without involving the canonical TGF-β pathway. This is in line with several reports that demonstrated a mTOR-I dose-related induction of CTGF at gene and protein levels in vitro and in vivo [49,50,51,52].

Moreover, Xu et al. have demonstrated that rapamycin, an analogue of EVE, exerted a profibrotic effect in lung epithelial cells as well as in lung fibroblasts via up-regulation of CTGF expression and PI3K/AKT pathway [50,51]. Similarly, Mikaelian et al. using a combination of RNAi and pharmacological approaches showed that inhibition of mTOR triggers EMT in mammalian epithelial cells by a mechanism TGF-β independent [53]. In the transplant context it has been described a synergistic fibrotic effect of sirolimus with cyclosporine in kidney also mediated by the up-regulation of CTGF [54,55].

Another interested gene up-regulated by EVE, selected by microarray and validated by RT-PCR, was metalloproteinase 12 (*MMP12*), a member of the zinc-dependent endopeptidases family able to proteolyze all components of the extracellular matrix [56,57] by degrading collagen, other extracellular filaments, cytokines, growth factors and their receptors. *MMP12* has a pivotal role in TGF-β mediated pulmonary fibrosis [58,59].

Interestingly, other identified genes by transcriptomic analysis and target of miR-8485 (Table 7) were Kallmann syndrome-1 gene (*KAL1*, fold change: 1.705), Limb-bud and heart (*LBH*, fold change: 1.808) and insulin receptor substrates 2 (*IRS2*, fold change: 1.646) that resulted up-regulated after 100 nM EVE treatment and Protocadherin 7 (*PCDH7*, fold change: −1.625) down-regulated by similar treatment. All of them have been described in literature as directly or indirectly involved in the EMT.

*KAL1*, codes for anosmin-1, a cell adhesion protein in extracellular matrix induced by TGF-β [60,61]. *IRS2* expression appears to repress the expression of E-cadherin [62], marker of epithelial cells deregulated during EMT.

*LBH* is a transcription cofactor with both transcriptional activator and corepressor functions. *LBH* is a direct Wnt/β-catenin target gene and is induced by TGF-β [63,64]. Wnt/β-catenin signaling activation occurs in cells during EMT [65] and treated with mTOR-I.

Protocadherin 7 is an integral membrane protein having a role in cell–cell recognition and adhesion. Down-regulation of *PCDH7* gene was correlated with E-cadherin inhibition [66].

All these findings, although speculatively interesting, need to be validated in vivo. Our study is an hypothesis generating study that should be considered a starting point for bio-molecular study involving transplanted patients or animal models.

Nevertheless, after 21 days in culture, most of the cells were not ciliated and we cannot exclude that differentiation state may have affected the response to EVE (Figure S3).

However, our results suggested that high concentrations of EVE, through the activation of a multi-factorial biological/cellular machinery, may lead to pulmonary fibrosis and underlined potential pathogenetic, diagnostic biomarkers and targets for future pharmacological interventions to introduce in the “day by day” clinical practice. Finally, at a clinical point of view, we confirm that, whenever possible, the dose of EVE should be the minimized in patients with early signs of lung toxicity.

## 4. Materials and Methods

### 4.1. Cell Culture Treatment

Primary wild-type bronchial epithelial cells (BE63/3 and BE121/3) were obtained from “Servizio Colture Primarie” of the Italian Cystic Fibrosis Research Foundation (ICFRF) and cultured following the supplier instructions [67]. The protocols to isolate, culture, store, and study bronchial epithelial cells from patients undergoing lung transplant was approved by the Ethical Committee of Gaslini Institute (ethical approval number IGG:192 date of approval: 9/24/2010) under the supervision of the Italian Ministry of Health. Cells were grown on rat tail collagen-coated tissue culture plates in serum-free LHC9/RPMI 1640 medium at 37 °C and 5% CO_2_.

After 4–5 passages, cells were seeded on Transwell porous inserts. After 24 h from seeding, the medium was switched to DMEM/F12 supplemented with 2% Ultroser G, 2 mM l-glutammine, 100 U/mL penicillin, 100 μg/mL streptomycin.

Exchange of culture medium is repeated every day on both sides of permeable supports up to 5 days. Then the apical culture medium was removed, and the medium was added only in the basolateral side (air-liquid interface) favoring a differentiation of the epithelium (Figure S3). After 11 days the epithelium was treated with EVE (5 nM and 100 nM) and TGF-β (20 ng/mL), an EMT inducer, for 24 h. “The timing of cell culture for gene expression and western blot experiments (17 days) was based on clear instructions supplied by the “Servizio Colture Primarie” of the ICFRF in order to reach the differentiation of epithelium”. Although the in vitro model cannot completely represent the in vivo pharmacokinetic/effect of this drug, we can postulate that 5 nM EVE corresponds to a trough level of approximately 5 ng/mL (drug level frequently reached in the immunosuppressive maintenance therapy of solid organ transplantation), while 100 nM may correspond to very high dosages (trough level more than 50 ng/mL) that patients could reach in anticancer therapy.

NIH/3T3 fibroblasts, purchased from American Type Culture Collection (Manassas, VA, USA) were maintained at 37 °C in DMEM supplemented with 10% FCS, 100 U/mL penicillin, 100 μg/mL streptomycin, and 2 mM l-glutamine. Cells were treated with or without 5 and 100 nM Everolimus for 24 h.

### 4.2. RNA Extraction and Gene Expression Profiling

Trizol reagent (Invitrogen) was used to extract total RNA and then, yield and purity were checked using a Nanodrop spectrophotometer.

Gene expression data were produced using the HumanHT-12 v3 Expression BeadChip (Release 38, Illumina, San Diego, CA, USA). Five hundred ng total RNA from BE63/3 was used to synthesize biotin-labeled cRNA using the Illumina^®^TotalPrep™ RNA amplification kit (Applied Biosystems, Foster City, CA, USA). Quality of labelled cRNA was assessed by NanoDrop^®^ ND-100 spectrophotometer and the Agilent 2100 Bioanalyzer. Then, 750 ng biotinylated cRNA was used for hybridization to illumina microarrays that were then scanned with the HiScanSQ.

### 4.3. Pathway Analysis

The Ingenuity Pathway Analysis software (IPA, Ingenuity System, Redwood City, CA, USA) was used to assess biological relationships among differentially regulated genes. The reference gene selection was performed by own software written in Java program language. The canonical pathways generated by IPA are the most significant for the uploaded data set. Fischer’s exact test with false discovery rate (FDR) option was used to calculate the significance of the canonical pathway.

### 4.4. MicroRNA Expression Profiling

Fluorescently-labeled miRNAs were generated using the miRNA Complete Labeling and Hybridization kit (Agilent Technologies, Santa Clara, CA, USA), with a sample input of 100 ng of total RNA from BE63/3 and hybridized for 20 h at 55 °C on the Agilent 8 × 60 K Human miRNA Microarray slide (Agilent Technologies), based on miRBase database (Release 21.0). Following hybridization, the slides were washed and scanned using the High-Resolution Microarray C Scanner (Agilent Technologies). The image files were processed using the Agilent Feature Extraction software (v10.7.3): the microarray grid was correctly placed; inlier pixels were identified, and outlier pixels were rejected.

### 4.5. Real-Time PCR

Five hundred ng total RNA from each sample was reverse transcribed into cDNA using the High Capacity cDNA Reverse Transcription Kit (Applied Biosystems). Real-time PCR amplification reactions were performed in duplicate via SYBR Green chemistry on CFX-connect (Bio-Rad, Hercules, CA, USA) and SsoAdvanced™ Universal SYBR^®^ Green Supermix (Bio-Rad). Primers for α-SMA, VIM, FN, MMP12, CTGF, CDH6, COL12A1, FAP, KAL1, LBH, PIM1 and glyceraldehyde-3-phosphate dehydrogenase (GAPDH) were obtained from Qiagen (QuantiTect Primer Assay, Hilden, Germany).

The comparative *C*_t_ method (ΔΔ*C*_t_) was used to quantify gene expression and the relative quantification was calculated as 2^−ΔΔ*C*t^. Melting curve analysis was employed to exclude non-specific amplification products.

### 4.6. Western Blot

Equal amounts of proteins were resolved in 10% SDS-PAGE and electrotransferred to nitrocellulose membranes. Non-specific binding was blocked for 1 h at room temperature with non-fat milk (5%) in TBST buffer (50 mM Tris-HCl, pH 7.4, 150 mM NaCl, 0.1% Tween 20). Membranes were exposed to primary antibodies directed against GAPDH (Santa Cruz sc-25778), CTGF (NovusBio, Littleton, CO, USA) and collagen1 (ORIGENE TA309096) (overnight at 4 °C) and incubated with a secondary peroxidase-conjugated antibody for 1 h at room temperature. The signal was detected with SuperSignals West Pico Chemiluminescent substrate solution (Pierce) according to the manufacturer’s instructions.

### 4.7. Transepithelial Resistance (TER)

Millicell-ERS ohmmeter with electrodes (Millipore) was used to measure TER (alternating current applied between the electrodes: ±20 μA and frequency: 12.5 Hz). The resistance of the monolayer multiplied by the effective surface area was used to obtain the electrical resistance of the monolayer (Ω cm^2^). Once stable resistances were obtained, different culture media (control, EVE 5 nM, EVE 100 nM, TGF-β 20 ng/mL) were tested. After the addition of test solutions, measurements were taken at 24 h.

### 4.8. Statistical Analysis

For transcriptomics statistical analyses were carried out by Genespring GX 11.0 software (Agilent Technologies). Gene probe sets were filtered based on the FDR method of Benjamini–Hochberg and fold-change. Only genes that were significantly (adjusted-*p* value < 0.05 and fold-change > 1.5) modulated were considered for further analysis.

In the miRNome analysis, after normalization (Quantile method), unpaired *t*-test (*p*-value cut-off: 0.05 and fold-change cut-off: 2.0, after Benjamini–Hochberg multiple testing correction) was employed to identify most differentially expressed probes.

For the statistical analysis of RT-PCR and western-blot, differences between control and treated cell were compared using Student’s *t*-test. A *p*-value < 0.05 was set as statistically significant.

## Figures and Tables

**Table 1:** List of the differentially expressed probe sets after treatment with 100 nM EVE.

Probe ID	Fold Change	Regulation	Symbol	Entrez Gene ID	Definition
4760626	2.275	Up	MMP12	4321	matrix metallopeptidase 12 (macrophage elastase), mRNA.
4780209	2.218	Up	MMP12	4321	matrix metallopeptidase 12 (macrophage elastase) mRNA.
670041	1.925	Up	AKAP12	9590	A kinase (PRKA) anchor protein (gravin) 12, transcript variant 2, mRNA.
6770746	1.903	Up	LOC728715	728715	similar to hCG38149 (LOC728715), mRNA.
4640086	1.814	Up	FOXQ1	94234	forkhead box Q1, mRNA.
2810246	1.808	Up	LBH	81606	limb bud and heart development homolog (mouse) (LBH), mRNA.
6330270	1.804	Up	GPC4	2239	glypican 4, mRNA.
6620201	1.789	Up	KLHL24	54800	kelch-like 24 (Drosophila), mRNA.
5690687	1.783	Up	CTGF	1490	connective tissue growth factor, mRNA.
5420577	1.775	Up	CLCA4	22802	chloride channel, calcium activated, family member 4, mRNA.
2640292	1.769	Up	CTGF	1490	connective tissue growth factor, mRNA.
1070477	1.753	Up	ALDH1A1	216	aldehyde dehydrogenase 1 family, member A1, mRNA.
3130301	1.729	Up	PIM1	5292	pim-1 oncogene, mRNA.
6620008	1.705	up	KAL1	3730	Kallmann syndrome 1 sequence, mRNA.
4040576	1.704	up	IL6	3569	interleukin 6 (interferon, beta 2), mRNA.
1820315	1.677	up	C4orf26	152816	chromosome 4 open reading frame 26 (C4orf26), mRNA.
1990142	1.671	up	C20orf114	92747	chromosome 20 open reading frame 114 (C20orf114), mRNA.
1940647	1.668	up	HBP1	26959	HMG-box transcription factor 1, mRNA.
2640324	1.665	up	SLC46A3	283537	solute carrier family 46, member 3, mRNA.
3800241	1.651	up	CDH6	1004	cadherin 6, type 2, K-cadherin (fetal kidney), mRNA.
6110736	1.646	up	IRS2	8660	insulin receptor substrate 2, mRNA.
4610056	1.641	up	FLRT2	23768	fibronectin leucine rich transmembrane protein 2, mRNA.
6420687	1.638	up	PLUNC	51297	palate, lung and nasal epithelium carcinoma associated, transcript variant 2, mRNA.
6420465	1.625	up	GABARAPL1	23710	GABA(A) receptor-associated protein like 1, mRNA.
4780128	1.625	up	ATF3	467	activating transcription factor 3, transcript variant 4, mRNA.
160242	1.622	up	C13orf15	28984	chromosome 13 open reading frame 15 (C13orf15), mRNA.
2650709	1.620	up	CDH11	1009	cadherin 11, type 2, OB-cadherin (osteoblast), mRNA.
2230767	1.615	up	LOC387825	387825	misc_RNA (LOC387825), miscRNA.
6860228	1.610	up	C5orf41	153222	chromosome 5 open reading frame 41 (C5orf41), mRNA.
6510754	1.609	up	ALDH1A1	216	aldehyde dehydrogenase 1 family, member A1, mRNA.
1980255	1.605	up	RNF39	80352	ring finger protein 39, transcript variant 2, mRNA.
6840491	1.604	up	C5orf41	153222	chromosome 5 open reading frame 41 (C5orf41), mRNA.
4280228	1.595	up	IVNS1ABP	10625	influenza virus NS1A binding protein, mRNA.
5080021	1.593	up	BIRC3	330	baculoviral IAP repeat-containing 3, transcript variant 1, mRNA.
6400131	1.589	up	CYP24A1	1591	cytochrome P450, family 24, subfamily A, polypeptide 1, nuclear gene encoding mitochondrial protein, mRNA.
7160239	1.580	up	FOSB	2354	FBJ murine osteosarcoma viral oncogene homolog B, mRNA.
380689	1.578	up	TSC22D1	8848	TSC22 domain family, member 1, transcript variant 1, mRNA.
3060095	1.574	up	COL12A1	1303	collagen, type XII, alpha 1, transcript variant short, mRNA.
1410209	1.571	up	SGK1	6446	serum/glucocorticoid regulated kinase 1, transcript variant 1, mRNA.
2190553	1.556	up	FZD6	8323	frizzled homolog 6 (Drosophila), mRNA.
4570075	1.544	up	KIAA1641	57730	KIAA1641, transcript variant 7, mRNA.
5090626	1.540	up	FAP	2191	fibroblast activation protein, alpha, mRNA.
6620538	1.540	up	UBL3	5412	ubiquitin-like 3, mRNA.
5960398	1.537	up	NT5E	4907	5′-nucleotidase, ecto (CD73), mRNA.
5570731	1.533	up	C8orf4	56892	chromosome 8 open reading frame 4 (C8orf4), mRNA.
830639	1.531	up	LOC653778	653778	similar to solute carrier family 25, member 37 (LOC653778), mRNA.
3290187	1.529	up	PCMTD1	115294	protein-l-isoaspartate (d-aspartate) O-methyltransferase domain containing 1 (PCMTD1), mRNA.
3440670	1.517	up	LOC402251	402251	similar to eukaryotic translation elongation factor 1 alpha 2 (LOC402251), mRNA.
630315	1.514	up	DHRS9	10170	dehydrogenase/reductase (SDR family) member 9, transcript variant 1, mRNA.
1410161	1.513	up	KLHL5	51088	kelch-like 5 (Drosophila), transcript variant 3, mRNA.
4150575	1.513	up	LETMD1	25875	LETM1 domain containing 1, transcript variant 2, mRNA.
7210497	1.513	up	NUAK1	9891	NUAK family, SNF1-like kinase, 1, mRNA.
1240440	1.511	up	TXNIP	10628	thioredoxin interacting protein, mRNA.
4760747	1.509	up	TPST1	8460	tyrosylprotein sulfotransferase 1, mRNA.
2360220	1.508	up	MATR3	9782	matrin 3, transcript variant 1, mRNA.
3800431	1.508	up	RCOR3	55758	REST corepressor 3, mRNA.
4390450	1.504	up	SGK	6446	serum/glucocorticoid regulated kinase, mRNA.
2450465	1.503	up	CYBRD1	79901	cytochrome b reductase 1, mRNA.
6110053	1.501	up	ZNF32	7580	zinc finger protein 32, transcript variant 2, mRNA.
4570398	1.501	up	F2R	2149	coagulation factor II (thrombin) receptor, mRNA.
3800050	−1.503	down	ADCY3	109	adenylate cyclase 3, mRNA.
5900008	−1.504	down	KLK11	11012	kallikrein-related peptidase 11, transcript variant 2, mRNA.
5080605	−1.504	down	SNRPA1	6627	small nuclear ribonucleoprotein polypeptide A′, mRNA.
4560541	−1.521	down	MLKL	197259	mixed lineage kinase domain-like, mRNA.
520682	−1.523	down	CPA4	51200	carboxypeptidase A4, mRNA.
4010296	−1.527	down	RNASE1	6035	ribonuclease, RNase A family, 1 (pancreatic), transcript variant 1, mRNA.
6350161	−1.530	down	LCP1	3936	lymphocyte cytosolic protein 1 (l-plastin), mRNA.
4730605	−1.532	down	AURKA	6790	aurora kinase A, transcript variant 5, mRNA.
6840075	−1.532	down	NP	4860	nucleoside phosphorylase, mRNA.
6770187	−1.533	down	SPRR2A	6700	small proline-rich protein 2A, mRNA.
870131	−1.533	down	HSPA5	3309	heat shock 70 kDa protein 5 (glucose-regulated protein, 78 kDa), mRNA.
1570193	−1.535	down	ARHGDIB	397	Rho GDP dissociation inhibitor (GDI) beta, mRNA.
2450167	−1.537	down	RPL29	6159	ribosomal protein L29, mRNA.
7510709	−1.540	down	CEP55	55165	centrosomal protein 55 kDa, mRNA.
2350465	−1.544	down	RPL29	6159	ribosomal protein L29, mRNA.
160097	−1.546	down	MELK	9833	maternal embryonic leucine zipper kinase, mRNA.
3930703	−1.547	down	WDR4	10785	WD repeat domain 4, transcript variant 2, mRNA.
1170066	−1.554	down	SULT2B1	6820	sulfotransferase family, cytosolic, 2B, member 1, transcript variant 1, mRNA.
2070520	−1.556	down	CDCA7	83879	cell division cycle associated 7, transcript variant 1, mRNA.
6550048	−1.559	down	DHCR7	1717	7-dehydrocholesterol reductase, mRNA.
5310634	−1.566	down	FASN	2194	fatty acid synthase, mRNA.
6560494	−1.566	down	ARTN	9048	artemin, transcript variant 2, mRNA.
5860348	−1.568	down	SC4MOL	6307	sterol-C4-methyl oxidase-like, transcript variant 2, mRNA.
5270112	−1.570	down	HMGCS1	3157	3-hydroxy-3-methylglutaryl-Coenzyme A synthase 1 (soluble), transcript variant 2, mRNA.
5690274	−1.571	down	MCM6	4175	minichromosome maintenance complex component 6, mRNA.
940487	−1.573	down	FUT3	2525	fucosyltransferase 3 (galactoside 3(4)-l-fucosyltransferase, Lewis blood group), transcript variant 4, mRNA.
5810154	−1.580	down	ALOX15B	247	arachidonate 15-lipoxygenase, type B, transcript variant b, mRNA.
870546	−1.581	down	MAD2L1	4085	MAD2 mitotic arrest deficient-like 1 (yeast), mRNA.
6020139	−1.588	down	KLK7	5650	kallikrein-related peptidase 7, transcript variant 1, mRNA.
4250156	−1.589	down	EBP	10682	emopamil binding protein (sterol isomerase), mRNA.
10341	−1.599	down	SHMT2	6472	serine hydroxymethyltransferase 2 (mitochondrial), nuclear gene encoding mitochondrial protein, mRNA.
5360678	−1.602	down	DHCR7	1717	7-dehydrocholesterol reductase, transcript variant 1, mRNA.
6580059	−1.610	down	UCP2	7351	uncoupling protein 2 (mitochondrial, proton carrier), nuclear gene encoding mitochondrial protein, mRNA.
5090278	−1.610	down	GPX2	2877	glutathione peroxidase 2 (gastrointestinal), mRNA.
3940673	−1.617	down	LOC728285	728285	similar to keratin associated protein 2-4 (LOC728285), mRNA.
2650564	−1.623	down	RARRES3	5920	retinoic acid receptor responder (tazarotene induced) 3, mRNA.
360367	−1.625	down	PCDH7	5099	protocadherin 7, transcript variant a, mRNA.
7560364	−1.635	down	LOC729779	729779	misc_RNA (LOC729779), miscRNA.
780528	−1.635	down	CKS2	1164	CDC28 protein kinase regulatory subunit 2, mRNA.
5960224	−1.636	down	PTTG3P	26255	pituitary tumor-transforming 3 (pseudogene), non-coding RNA.
4730196	−1.653	down	TK1	7083	thymidine kinase 1, soluble, mRNA.
1510296	−1.656	down	ASNS	440	asparagine synthetase, transcript variant 1, mRNA.
1190142	−1.657	down	EMILIN2	84034	elastin microfibril interfacer 2, mRNA.
1170170	−1.662	down	STC2	8614	stanniocalcin 2, mRNA.
2140128	−1.670	down	SCD	6319	stearoyl-CoA desaturase (delta-9-desaturase), mRNA.
5360070	−1.674	down	CCNB2	9133	cyclin B2, mRNA.
3990619	−1.675	down	TOP2A	7153	topoisomerase (DNA) II alpha 170 kDa, mRNA.
3780047	−1.679	down	GBP6	163351	guanylate binding protein family, member 6, mRNA.
2000148	−1.683	down	IFIT1	3434	interferon-induced protein with tetratricopeptide repeats 1, transcript variant 2, mRNA.
2070494	−1.700	down	PRC1	9055	protein regulator of cytokinesis 1, transcript variant 2, mRNA.
10414	−1.704	down	PTTG1	9232	pituitary tumor-transforming 1, mRNA.
2940110	−1.720	down	UHRF1	29128	ubiquitin-like with PHD and ring finger domains 1, transcript variant 1, mRNA.
1510291	−1.733	down	PTTG1	9232	pituitary tumor-transforming 1, mRNA.
1780446	−1.739	down	PCK2	5106	phosphoenolpyruvate carboxykinase 2 (mitochondrial), nuclear gene encoding mitochondrial protein, transcript variant 1, mRNA.
1660521	−1.745	down	SPRR2D	6703	small proline-rich protein 2D, mRNA.
730689	−1.763	down	LOC652595	652595	similar to U2 small nuclear ribonucleoprotein A (U2 snRNP-A) (LOC652595), mRNA.
5090754	−1.766	down	KIAA0101	9768	KIAA0101, transcript variant 1, mRNA.
5080139	−1.789	down	PRSS3	5646	protease, serine, 3 (mesotrypsin), mRNA.
3800452	−1.805	down	EMP3	2014	epithelial membrane protein 3, mRNA.
1230047	−1.810	down	CBS	875	cystathionine-beta-synthase, mRNA.
6370615	−1.858	down	TGM1	7051	transglutaminase 1 (K polypeptide epidermal type I, protein-glutamine-gamma-glutamyltransferase), mRNA.
5310471	−1.894	down	UBE2C	11065	ubiquitin-conjugating enzyme E2C, transcript variant 6, mRNA.
7380719	−1.897	down	IGFBP6	3489	insulin-like growth factor binding protein 6, mRNA.
940327	−1.907	down	KLK13	26085	kallikrein-related peptidase 13, mRNA.
520195	−1.914	down	TMEM79	84283	transmembrane protein 79, mRNA.
4040398	−1.954	down	MAL	4118	mal, T-cell differentiation protein, transcript variant d, mRNA.
1990630	−1.979	down	TRIB3	57761	tribbles homolog 3 (Drosophila), mRNA.
430446	−1.996	down	KRT81	3887	keratin 81, mRNA.
4260368	−2.022	down	UBE2C	11065	ubiquitin-conjugating enzyme E2C, transcript variant 3, mRNA.
290767	−2.038	down	KRTDAP	388533	keratinocyte differentiation-associated protein, mRNA.
6520139	−2.046	down	FGFR3	2261	fibroblast growth factor receptor 3 (achondroplasia, thanatophoric dwarfism), transcript variant 2, mRNA.
620102	−2.046	down	MALL	7851	mal, T-cell differentiation protein-like, mRNA.
5870653	−2.050	down	LOC651397	651397	misc_RNA (LOC651397), miscRNA.
4050398	−2.071	down	KLK12	43849	kallikrein-related peptidase 12, transcript variant 1, mRNA.
7330753	−2.102	down	ACAT2	39	acetyl-Coenzyme A acetyltransferase 2, mRNA.
4900458	−2.147	down	KRT14	3861	keratin 14 (epidermolysis bullosa simplex, Dowling-Meara, Koebner), mRNA.
540546	−2.283	down	KRT4	3851	keratin 4, mRNA.
1500010	−2.322	down	CDC20	991	cell division cycle 20 homolog (*S. cerevisiae*), mRNA.
6550356	−2.430	down	SPRR2C	6702	small proline-rich protein 2C (pseudogene), non-coding RNA.
4850674	−2.452	down	PSAT1	29968	phosphoserine aminotransferase 1, transcript variant 2, mRNA.
5890400	−2.577	down	SPRR2E	6704	small proline-rich protein 2E, mRNA.
240086	−2.608	down	PHGDH	26227	phosphoglycerate dehydrogenase, mRNA.
7650441	−2.696	down	FGFBP1	9982	fibroblast growth factor binding protein 1, mRNA.
5810546	−2.894	down	SPRR2E	6704	small proline-rich protein 2E, mRNA.
7330184	−2.933	down	SPRR1A	6698	small proline-rich protein 1A, mRNA.
2230035	−2.936	down	KRT13	3860	keratin 13, transcript variant 2, mRNA.
4610131	−3.284	down	SPRR3	6707	small proline-rich protein 3, transcript variant 1, mRNA.

In red up-regulated and in green down-regulated genes in BE63/3 cells treated with 100 nM EVE compared to CTR.

**Table 2:** List of pathways differentially regulated after 100 nM EVE.

Pathways	Adj. *p* Value	Associated Genes
Epidermis development	1.24 × 10^−6^	*ALOX15B, CTGF, FOXQ1, FZD6, KLK7, KRT14, RNASE1, SPRR1A, SPRR2A, SPRR2D, SPRR2E, SPRR3, TGM1, TMEM79, TXNIP*
Keratinization	5.22 × 10^−6^	*SPRR1A, SPRR2A, SPRR2D, SPRR2E, SPRR3, TGM1, TMEM79*
Negative regulation of cell division	2.58 × 10^−5^	*CDC20, FGFR3, MAD2L1, PTTG1, PTTG3P, RGCC, TXNIP, UBE2C*
Negative regulation of mitotic nuclear division	2.81 × 10^−5^	*CDC20, FGFR3, MAD2L1, PTTG1, PTTG3P, RGCC, UBE2C*
Keratinocyte differentiation	3.05 × 10^−5^	*ALOX15B, SPRR1A, SPRR2A, SPRR2D, SPRR2E, SPRR3, TGM1, TMEM79, TXNIP*
L-serine metabolic process	3.54 × 10^−5^	*CBS, PHGDH, PSAT1, SHMT2*
Epidermal cell differentiation	9.21 × 10^−5^	*ALOX15B, RNASE1, SPRR1A, SPRR2A, SPRR2D, SPRR2E, SPRR3, TGM1, TMEM79, TXNIP*
L-serine biosynthetic process	9.75 × 10^−5^	*PHGDH, PSAT1, SHMT2*
Negative regulation of nuclear division	1.10 × 10^−4^	*CDC20, FGFR3, MAD2L1, PTTG1, PTTG3P, RGCC, UBE2C*
Skin development	1.82 × 10^−4^	*ALOX15B, FOXQ1, FZD6, SPRR1A, SPRR2A, SPRR2D, SPRR2E, SPRR3, TGM1, TMEM79, TXNIP*
Peptide cross-linking	2.05 × 10^−4^	*SPRR1A, SPRR2A, SPRR2D, SPRR2E, SPRR3, TGM1*
Serine family amino acid biosynthetic process	3.55 × 10^−4^	*CBS, PHGDH, PSAT1, SHMT2*
Regulation of collagen metabolic process	5.84 × 10^−4^	*CTGF, F2R, FAP, IL6, RGCC*
Regulation of multicellular organismal metabolic process	6.51 × 10^−4^	*CTGF, F2R, FAP, IL6, RGCC*
Steroid biosynthesis	6.77 × 10^−4^	*CYP24A1, DHCR7, EBP, MSMO1*
Chromosome separation	0.00192	*CDC20, MAD2L1, PTTG1, PTTG3P, TOP2A, UBE2C*
Negative regulation of mitotic sister chromatid separation	0.00199	*CDC20, MAD2L1, PTTG1, PTTG3P, UBE2C*
Collagen metabolic process	0.00200	*COL12A1, CTGF, F2R, FAP, IL6, MMP12, RGCC*
Negative regulation of mitotic sister chromatid segregation	0.00231	*CDC20, MAD2L1, PTTG1, PTTG3P, UBE2C*
Multicellular organismal macromolecule metabolic process	0.00248	*COL12A1, CTGF, F2R, FAP, IL6, MMP12, RGCC*
Negative regulation of sister chromatid segregation	0.00267	*CDC20, MAD2L1, PTTG1, PTTG3P, UBE2C*
Negative regulation of chromosome segregation	0.00267	*CDC20, MAD2L1, PTTG1, PTTG3P, UBE2C*
Regulation of nuclear division	0.00302	*AURKA, CDC20, FGFR3, MAD2L1, PTTG1, PTTG3P, RGCC, UBE2C*
Multicellular organismal metabolic process	0.00456	*COL12A1, CTGF, F2R, FAP, IL6, MMP12, RGCC*
Regulation of collagen biosynthetic process	0.00457	*CTGF, F2R, IL6, RGCC*
Mitotic sister chromatid separation	0.00664	*CDC20, MAD2L1, PTTG1, PTTG3P, UBE2C*
Regulation of mitotic sister chromatid segregation	0.00834	*CDC20, MAD2L1, PTTG1, PTTG3P, UBE2C*
Sister chromatid segregation	0.00851	*CDC20, CEP55, MAD2L1, PTTG1, PTTG3P, TOP2A, UBE2C*
Glycine, serine and threonine metabolism	0.00873	*CBS, PHGDH, PSAT1, SHMT2*
Collagen biosynthetic process	0.00873	*CTGF, F2R, IL6, RGCC*
Oocyte meiosis	0.01153	*ADCY3, AURKA, CCNB2, CDC20, MAD2L1, PTTG1*
Regulation of sister chromatid segregation	0.01277	*CDC20, MAD2L1, PTTG1, PTTG3P, UBE2C*
Negative regulation of chromosome organization	0.01396	*ARTN, CDC20, MAD2L1, PTTG1, PTTG3P, UBE2C*
PERK-mediated unfolded protein response	0.01404	*ASNS, ATF3, HSPA5*
Regulation of stress fiber assembly	0.01630	*CTGF, RGCC, RNASE1*
FoxO signaling pathway	0.01634	*CCNB2, GABARAPL1, IL6, IRS2, PCK2, SGK1*
Anaphase-promoting complex-dependent proteasomal ubiquitin-dependent protein catabolic process	0.01664	*AURKA, CDC20, MAD2L1, PTTG1, UBE2C*
Alpha-amino acid biosynthetic process	0.01664	*ASNS, CBS, PHGDH, PSAT1, SHMT2*
Positive regulation of collagen biosynthetic process	0.02234	*CTGF, F2R, RGCC*
Regulation of systemic arterial blood pressure by circulatory renin-angiotensin	0.02412	*CPA4, F2R, MMP12*
Positive regulation of multicellular organismal metabolic process	0.02412	*CTGF, F2R, RGCC*
Secondary alcohol biosynthetic process	0.02578	*DHCR7, EBP, HMGCS1, MSMO1*
Regulation of chromosome segregation	0.02590	*CDC20, MAD2L1, PTTG1, PTTG3P, UBE2C*
Negative regulation of proteasomal ubiquitin-dependent protein catabolic process	0.03145	*CDC20, MAD2L1, UBE2C*

In red up-regulated and in green down-regulated genes in BE63/3 cells treated with 100 nM EVE compared to CTR.

**Table 3:** List of probe sets differentially expressed after treatment with 5 nM EVE.

Probe ID	Fold Change	Regulation	Symbol	Entrez Gene ID	Definition
2230035	7.508	up	KRT13	3860	keratin 13, transcript variant 2, mRNA.
6510754	3.841	up	ALDH1A1	216	aldehyde dehydrogenase 1 family, member A1, mRNA.
1070477	3.395	up	ALDH1A1	216	aldehyde dehydrogenase 1 family, member A1, mRNA.
540546	2.749	up	KRT4	3851	keratin 4, mRNA.
1990142	2.644	up	C20orf114	92747	chromosome 20 open reading frame 114, mRNA.
5900368	2.385	up	MSMB	4477	microseminoprotein, beta-, transcript variant PSP94, mRNA.
4610131	2.358	up	SPRR3	6707	small proline-rich protein 3, transcript variant 1, mRNA.
3190110	2.194	up	MSMB	4477	microseminoprotein, beta-, transcript variant PSP94, mRNA.
630315	2.151	up	DHRS9	10170	dehydrogenase/reductase (SDR family) member 9, transcript variant 1, mRNA.
5420577	2.149	up	CLCA4	22802	chloride channel, calcium activated, family member 4, mRNA.
5560369	2.107	up	ALDH3A1	218	aldehyde dehydrogenase 3 family, memberA1, mRNA.
4150598	1.990	up	MSMB	4477	microseminoprotein, beta-, transcript variant PSP57, mRNA.
1820414	1.897	up	ATP12A	479	ATPase, H^+^/K^+^ transporting, nongastric, alpha polypeptide, mRNA.
3520709	1.888	up	ADH7	131	alcohol dehydrogenase 7 (class IV), mu or sigma polypeptide, mRNA.
7160468	1.807	up	DHRS9	10170	dehydrogenase/reductase (SDR family) member 9, transcript variant 1, mRNA.
5310646	1.795	up	AKR1B10	57016	aldo-keto reductase family 1, member B10 (aldose reductase), mRNA.
4250092	1.749	up	C10orf99	387695	chromosome 10 open reading frame 99, mRNA.
110372	1.748	up	CSTA	1475	cystatin A (stefin A), mRNA.
3710671	1.712	up	KRT15	3866	keratin 15, mRNA.
1770603	1.705	up	TCN1	6947	transcobalamin I (vitamin B12 binding protein, R binder family), mRNA.
6100537	1.655	up	FAM3D	131177	family with sequence similarity 3, member D, mRNA.
4540400	1.623	up	CYP4B1	1580	cytochrome P450, family 4, subfamily B, polypeptide 1, transcript variant 2, mRNA.
2900050	1.611	up	GSTA1	2938	glutathione S-transferase alpha 1, mRNA.
1510170	1.565	up	NLRP2	55655	NLR family, pyrin domain containing 2, mRNA.
5820400	1.526	up	CYP4B1	1580	cytochrome P450, family 4, subfamily B, polypeptide 1, mRNA.
130561	1.525	up	GSTA4	2941	glutathione S-transferase A4, mRNA.
3850246	1.513	up	HOPX	84525	HOP homeobox, transcript variant 3, mRNA.
7200612	−1.522	down	LOC730417	730417	hypothetical protein LOC730417, mRNA.
1510296	−1.556	down	ASNS	440	asparagine synthetase, transcript variant 1, mRNA.
3290390	−1.563	down	LOC729841	729841	misc_RNA, miscRNA.
7380193	−1.574	down	ARPC3	10094	actin related protein 2/3 complex, subunit 3, 21 kDa, mRNA.
130717	−1.610	down	ARPC1B	10095	actin related protein 2/3 complex, subunit 1B, 41 kDa, mRNA.
430446	−1.689	down	KRT81	3887	keratin 81, mRNA.

In red up-regulated and in green down-regulated genes in BE63/3 cells treated with 5 nM EVE compared to CTR.

**Table 4:** List of pathways differentially regulated after treatment with 5 nM EVE.

PATHWAYS	Adj. *p* Value	Associated Genes Found
Retinol metabolism	8.58 × 10^−5^	ADH7, ALDH1A1, DHRS9
Metabolism of xenobiotics by cytochrome P450	1.48 × 10^−5^	ADH7, ALDH3A1, GSTA1, GSTA4
Drug metabolism	1.37 × 10^−5^	ADH7, ALDH3A1, GSTA1, GSTA4
Retinoid metabolic process	1.41 × 10^−5^	ADH7, AKR1B10, ALDH1A1, DHRS9
Chemical carcinogenesis	1.96 × 10^−5^	ADH7, ALDH3A1, GSTA1, GSTA4
Cellular aldehyde metabolic process	2.60 × 10^−5^	ADH7, AKR1B10, ALDH1A1, ALDH3A1
Primary alcohol metabolic process	3.30 × 10^−6^	ADH7, AKR1B10, ALDH1A1, DHRS9
Retinol metabolic process	1.99 × 10^−5^	ADH7, ALDH1A1, DHRS9

In red up-regulated genes in BE63/3 cells treated with 5 nM EVE compared to CTR.

**Table 5:** List of microRNAs differentially regulated after treatment with 100 nM EVE.

Systematic Name	Regulation	Fold Change
hsa-miR-8485	up	5.372
hsa-miR-937-5p	up	1.787
hsa-miR-5194	up	1.694

**Table 6:** List of microRNAs differentially regulated after treatment with 5 nM EVE.

Systematic Name	Regulation	Fold Change
hsa-miR-8485	up	9.183
hsa-miR-4730	up	2.900
hsa-miR-5194	up	2.732
hsa-miR-6716-3p	up	2.561

**Table 7:** miRNA/mRNA pairs matched on the basis of mRNA and miRNA profiling results.

Cell Treatments	miRNA	Fold Change	mRNA Target	Gene Name
EVE 5 nM	miR-8485	9.183	CYP4B1	cytochrome P450, family 4, subfamily B, polypeptide 1
	miR-5194	2.732	ARPC3	actin related protein 2/3 complex, subunit 3, 21 kDa
EVE 100 nM	miR-8485	5.372	CYP24A1	cytochrome P450, family 24, subfamily A, polypeptide 1
			KAL1	Kallmann syndrome 1 sequence
			UBL3	ubiquitin-like 3
			IRS2	insulin receptor substrate 2
			CTGF	connective tissue growth factor
			LBH	limb bud and heart development
			FLRT2	fibronectin leucine rich transmembrane protein 2
			CDH6	cadherin 6, type 2, K-cadherin (fetal kidney)
			CYBRD1	cytochrome b reductase 1
			LETMD1	LETM1 domain containing 1
			FGFR3	fibroblast growth factor receptor 3
			CPA4	carboxypeptidase A4
			AURKA	aurora kinase A
			CBS	cystathionine-beta-synthase
			MAD2L1	MAD2 mitotic arrest deficient-like 1 (yeast)
			ADCY3	adenylate cyclase 3
			TMEM79	transmembrane protein 79
			IFIT1	interferon-induced protein with tetratricopeptide repeats 1
			PTTG1	pituitary tumor-transforming 1
			PCDH7	protocadherin 7
	miR-937-5p	1.787	CDH6	cadherin 6, type 2, K-cadherin (fetal kidney)
			KIAA0101	KIAA0101
			EMILIN2	elastin microfibril interfacer 2
	miR-5194	1.694	KLHL24	kelch-like family member 24
			FAP	fibroblast activation protein, alpha
			LBH	limb bud and heart development
			PIM1	pim-1 oncogene
			FLRT2	fibronectin leucine rich transmembrane protein 2
			LETMD1	LETM1 domain containing 1
			FGFR3	fibroblast growth factor receptor 3
			KIAA0101	KIAA0101
			RARRES3	retinoic acid receptor responder (tazarotene induced) 3
			ARTN	artemin
			IGFBP6	insulin-like growth factor binding protein 6
			LCP1	lymphocyte cytosolic protein 1 (L-plastin)
			MALL	small integral membrane protein 5
			SCD	LSM14B, SCD6 homolog B (*S. cerevisiae*)
			IFIT1	interferon-induced protein with tetratricopeptide repeats 1

In red up-regulated and in green down-regulated genes in BE63/3 cells treated with EVE (5 or 100 nM) compared to CTR.

## Supplementary Materials

Supplementary materials can be found at http://www.mdpi.com/1422-0067/19/4/1250/s1.
