# Supplementary figures and images for: In Vitro Identification of New Transcriptomic and miRNomic Profiles Associated with Pulmonary Fibrosis Induced by High Doses Everolimus: Looking for New Pathogenetic Markers and Therapeutic Targets
Source: Int J Mol Sci. 2018 Apr 20;19(4):1250. doi: 10.3390/ijms19041250 (PMC5979287; doi:10.3390/ijms19041250)

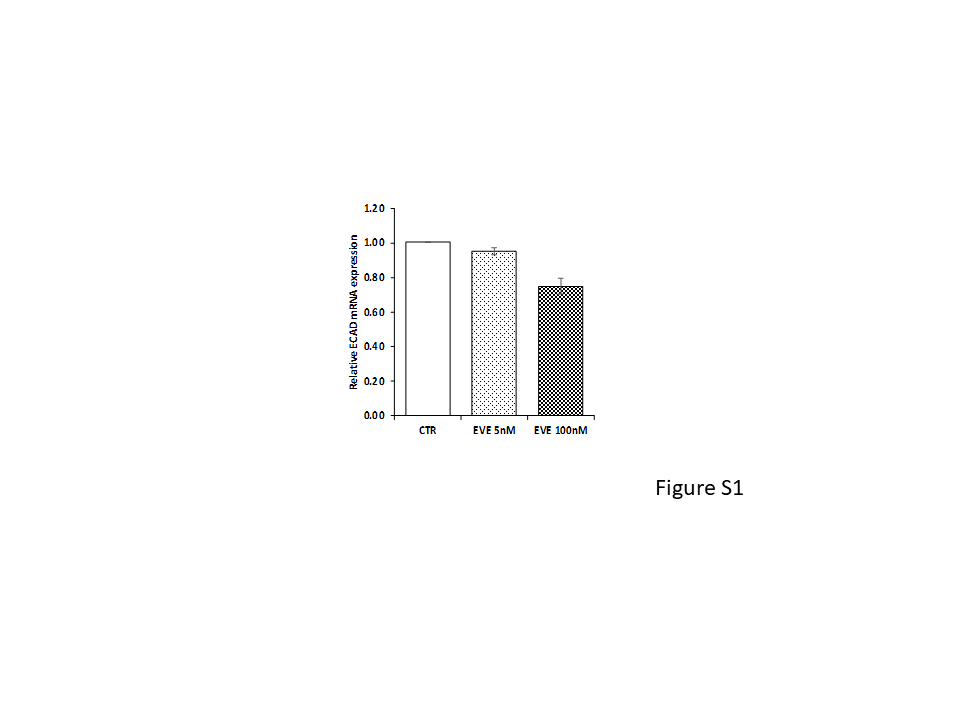

Supplement: Supplementary file 1 [file ijms-19-01250-s001.zip › FIGURE S1.tif]

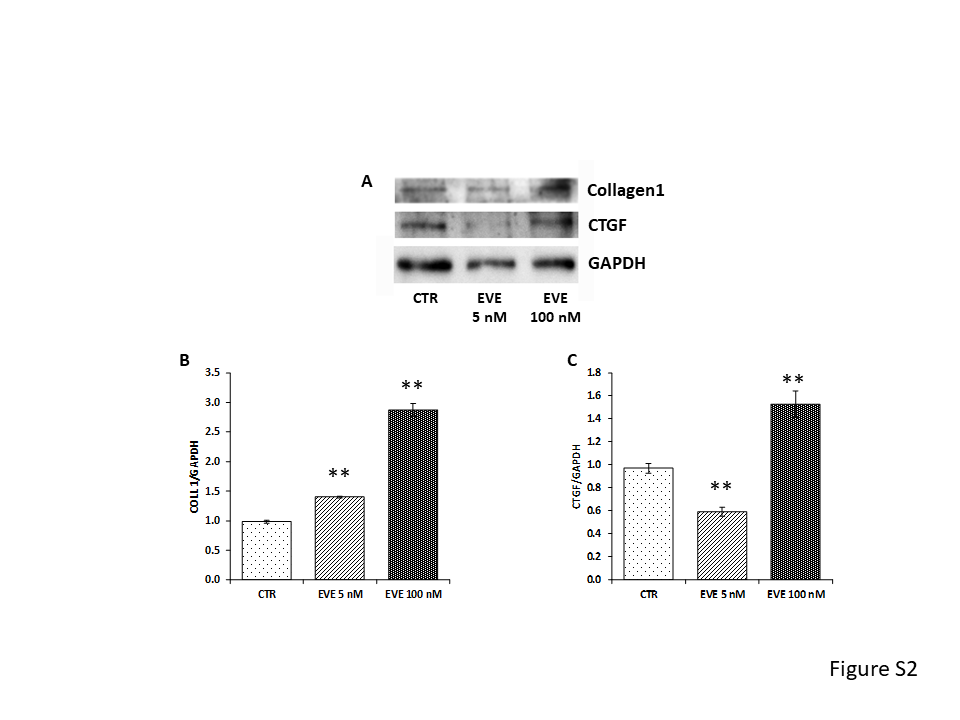

Supplement: Supplementary file 1 [file ijms-19-01250-s001.zip › FIGURE S2.tif]

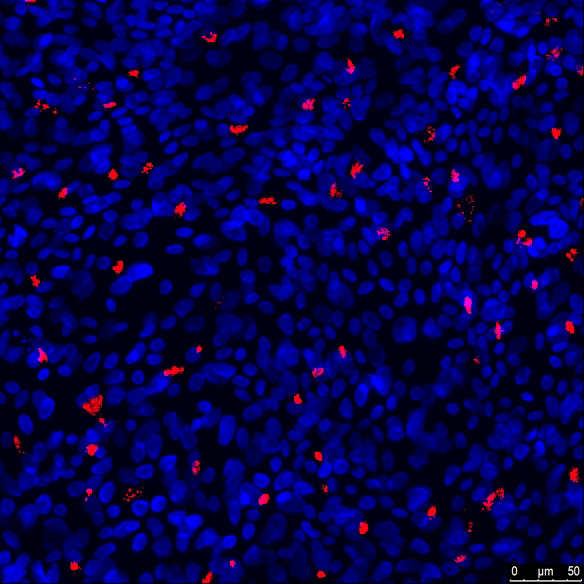

Supplement: Supplementary file 1 [file ijms-19-01250-s001.zip › FIGURE S3.tif]
