# Supplementary material for: In Vitro Identification of New Transcriptomic and miRNomic Profiles Associated with Pulmonary Fibrosis Induced by High Doses Everolimus: Looking for New Pathogenetic Markers and Therapeutic Targets
Source: Int J Mol Sci. 2018 Apr 20;19(4):1250. doi: 10.3390/ijms19041250 (PMC5979287; doi:10.3390/ijms19041250)
Supplement: Supplementary file 1 [file ijms-19-01250-s001.zip › Granata et al Supplementary File.doc]

**Supplementary material**

**In Vitro Identification of New Transcriptomic and miRNomic Profiles Associated With Pulmonary Fibrosis Induced by High Doses Everolimus: Looking For New Pathogenetic Marker and Therapeutic Targets.**

Simona Granata^1^, Gloria Santoro^1^, Valentina Masola^1^, Paola Tomei^1^, Fabio Sallustio^2,3^, Paola Pontrelli^2^, Matteo Accetturo^2^, Nadia Antonucci^1^, Pierluigi Carratù^4^, Antonio Lupo^1^, Gianluigi Zaza^1^.

^1^Renal Unit, Department of Medicine, University of Verona, Piazzale Stefani 1, 37126, Verona, Italy;

^2^University of Bari, Department of Emergency and Organ Transplantation, Piazza Giulio Cesare 11, 70124, Bari, Italy;

^3^Department of Basic Medical Sciences, Neuroscience and Sense Organs, University of Bari "Aldo Moro", Bari, Italy.

^4^Department of Respiratory Diseases, University of Bari "Aldo Moro", Piazza Giulio Cesare 11, 70124, Bari, Italy.

**Supplementary Methods**

*Hepatic stellate cells isolation and treatments*

Hepatic stellate cells (HSC) were isolated from normal male Winstar rats. All animals were purchased from Charles River Laboratories and were housed in the Animal Research Facility of Biological-Chemistry Department. They were maintained under a 12-h light–dark cycle and given rat chow and water ad libitum. The animal investigation was in accordance with the National Institute of Health guidelines for the care of Laboratory animals and was approved by the local committee for supervision of animal experiments at the University Hospital of Padova, Italy. This is over and above adherence to general guidelines.

HSCs were isolated from normal livers according to a modified Zhang method [Zhang X, Yu WP, Gao L, Wei KB, Ju JL, Xu JZ. Effects of lipopolysaccharides stimulated Kupffer cells on activation of rat hepatic stellate cells. World J Gastroenterol. 2004;10:610–3]; livers were perfused with a calcium-free buffer solution to wash out the blood, subsequently with a wash solution (calcium-free solution with CaCl_2_ 0.294 g/L, MgSO_4_ 0.097 g/L) and finally incubated in a digestion buffer (collagenase 0.6 g/L in Gey’s Balanced Salt Solution with Ca^2+^and Mg^2+^, pH 7.5) for 1 h (37°C, CO_2_ 5 %) to digest the extracellular matrix. The organs were cut into small pieces and subjected to homogenization to produce a single cell-suspension, which was centrifuged at 1450 × g for 18 min in 12 % (wt/vol) Nycodenz gradient to obtain a pure HSC fraction. Both the number and viability of HSCs were determined using the trypan blue dye exclusion test.

Collected HSCs were washed with Hank’s balanced salt solution and resuspended at a concentration of 1 × 105 cells/ml, in DMEM (supplemented with 20 % FBS and 1× antibiotic solution) and cultured on collagen type I coated well plates (10 μg/ml) in a 5 % CO2-humidified atmosphere. Serum-starved cells were subsequently detached for experiments using trypsin/EDTA solution.

All cell experiments were performed between passages 3 and 4.

HSC cells were plated in complete growth medium and then starved for 24 h in serum free medium. Cells were cultured for additional 24 h with 5 or 100 nM EVE and then assayed for gene and protein expression.

*Real-Time PCR for E-Cadherin*

Real-time PCR amplification reactions were performed in duplicate via SYBR Green chemistry on CFX-connect (Bio-Rad) and SsoAdvanced™ Universal SYBR® Green Supermix (Bio-Rad). Primers for E-Cadherin and GAPDH were obtained from Qiagen (QuantiTect Primer Assay).

The comparative Ct method (ΔΔCt) was used to quantify gene expression and the relative quantification was calculated as 2^-ΔΔCt^. The presence of non-specific amplification products was excluded by melting curve analysis.

*Immunofluorescence*

Bronchial epithelial cells cultured on air/liquid interface for 21 days were fixed in 4% paraformaldehyde. Filters were then excised from the support, permeabilized and saturated with 3% BSA in PBS. Then they were incubated overnight at 4°C with anti-acetylated tubulin antibody (Sigma T7451) diluted in 1% BSA in PBS for 1 h at room temperature. After being washed three times with PBS, the filters were incubated for 1 h at room temperature with the secondary antibody anti mouse-Alexa-546 (Thermo-Fisher). Cell nuclei were visualized with a Hoechst 33258. Images were obtained with a confocal LeicaSP5 microscope.

**Supplementary Results**

**Figure S1.** **Gene expression of E-CADHERIN.** Relative E-CAD expression evaluated by Real-time PCR in BE 63/3 cells treated or untreated with EVE (5 and 100 nM) expression values were normalized to GAPDH. Mean±S.D. (error bars) of three separate experiments performed in triplicate. High dosage EVE caused a downstream downregulation of E-CAD but it is not statistically significant (p=0.057).

**Figure S2. Protein expression of Collagen1 and CTGF in hepatic stellate cells.** (A) Representative western blotting experiments for Collagen1 and CTGF. Histograms represent the mean±SD of (B) Collagen1 and (C) CTGF protein level. GAPDH was included as loading control. **p<0.001 vs. CTR.

**Figure S3. Immunofluorescence staining of ciliary axoneme.** BE121/3 cells were cultured on air/liquid interface for 21 days and then stained with anti-acetylated tubulin antibody (red). Nuclei were stained with Hoechst 33258 (blue).
